# Dapagliflozin add-on to metformin in type 2 diabetes inadequately controlled with metformin: a randomized, double-blind, placebo-controlled 102-week trial

**DOI:** 10.1186/1741-7015-11-43

**Published:** 2013-02-20

**Authors:** Clifford J Bailey, Jorge L Gross, Delphine Hennicken, Nayyar Iqbal, Traci A Mansfield, James F List

**Affiliations:** 1 Life and Health Sciences, Aston University, Birmingham B4 7ET, UK; 2 Endocrine Division, Universidade Federal do Rio Grande do Sul, Avenida Paulo Gama 110-Reitoria, Porto Alegre, 90040-060, Brazil; 3 Global Biometric Services, Bristol-Myers Squibb, Parc de l'Alliance, Avenue de Finlande 4, Braine I'Alleud, B-1420, Belgium; 4 Global Clinical Research, Bristol-Myers Squibb, Route 206 & Province Line Road, Princeton, New Jersey, 08543, USA

**Keywords:** Dapagliflozin, metformin, SGLT2, sodium-glucose cotransporter 2, glycemic control, type 2 diabetes

## Abstract

**Background:**

Management of type 2 diabetes with metformin often does not provide adequate glycemic control, thereby necessitating add-on treatment. In a 24-week clinical trial, dapagliflozin, an investigational sodium glucose cotransporter 2 inhibitor, improved glycemic control in patients inadequately controlled with metformin. The present study is an extension that was undertaken to evaluate dapagliflozin as long-term therapy in this population.

**Methods:**

This was a long-term extension (total 102 weeks) of a 24-week phase 3, multicenter, randomized, placebo-controlled, double-blind, parallel-group trial. Patients were randomly assigned (1:1:1:1) to blinded daily treatment (placebo, or dapagliflozin 2.5 to 5, or 10 mg) plus open-label metformin (≥1,500 mg). The previously published primary endpoint was change from baseline in glycated hemoglobin (HbA1c) at 24 weeks. This paper reports the follow-up to week 102, with analysis of covariance model performed at 24 weeks with last observation carried forward; a repeated measures analysis was utilized to evaluate changes from baseline in HbA1c, fasting plasma glucose (FPG), and weight.

**Results:**

A total of 546 patients were randomized to 1 of the 4 treatments. The completion rate for the 78-week double-blind extension period was lower for the placebo group (63.5%) than for the dapagliflozin groups (68.3% to 79.8%). At week 102, mean changes from baseline HbA1c (8.06%) were +0.02% for placebo compared with -0.48% (*P *= 0.0008), -0.58% (*P *<0.0001), and -0.78% (*P *<0.0001) for dapagliflozin 2.5 to 5, and 10 mg, respectively. In addition, all dapagliflozin groups had sustained reductions from baseline in FPG (-1.07 to -1.47 mmol/l) and body weight (-1.10 to -1.74 kg) at 102 weeks, whereas increases were noted in placebo-treated patients for both of these outcomes. Events of hypoglycemia were rare and were not severe. Evidence suggestive of genital infection was reported in 11.7% to 14.6% of dapagliflozin patients and 5.1% of placebo patients, with one related discontinuation (dapagliflozin 5 mg). Evidence suggestive of urinary tract infection was reported in 8.0% to 13.3% of dapagliflozin patients and 8.0% of placebo patients, with one related discontinuation (dapagliflozin 2.5 mg).

**Conclusions:**

Dapagliflozin added to metformin for 102 weeks enabled sustained reductions in HbA1c, FPG, and weight without increased risk of hypoglycemia in patients with type 2 diabetes who were inadequately controlled on metformin alone.

**Trial registration:**

ClinicalTrials.gov: NCT00528879

## Background

Treatment of type 2 diabetes often begins with lifestyle management and/or metformin [1]. As β-cell function declines in the presence of insulin resistance, this makes maintenance of glycemic control challenging and usually necessitates add-on therapies. Because metformin acts to improve insulin sensitivity [2], the addition of therapy utilizing an insulin-independent pathway may be advantageous.

Inhibition of sodium-glucose cotransporter 2 (SGLT2) represents a novel approach to reduce hyperglycemia independently of insulin secretion or action [3-6]. SGLT2, located in the renal proximal tubule, reabsorbs most of the filtered glucose [7] and its inhibition represents a new pharmacotherapy for the treatment of type 2 diabetes. Dapagliflozin, a potent and selective SGLT2 inhibitor, has been shown to improve glycemic control in patients with type 2 diabetes when used as monotherapy [8] or in combination with metformin [9], sulfonylureas [10,11], thiazolidinedione [12], or insulin [6,13].

When dapagliflozin was added to metformin for 24 weeks in patients inadequately controlled on metformin alone (glycated hemoglobin (HbA1c) ≥7% and ≤10%), there was a dose-related mean reduction in HbA1c by -0.67% with dapagliflozin 2.5 mg, -0.70% with dapagliflozin 5 mg, and -0.84% with dapagliflozin 10 mg compared to -0.3% with placebo [9]. The present report describes a long-term double-blind extension of this study to examine the effectiveness and safety of dapagliflozin add-on to metformin for 102 weeks in patients with type 2 diabetes inadequately controlled on metformin monotherapy.

## Methods

A detailed description of the 24-week methods was published previously [9]. The 78-week extension is described below. This double-blind, parallel-group, placebo-controlled multicenter study recruited patients from 80 sites in Argentina, Brazil, Canada, Mexico, and USA. The study was performed in accordance of the Declaration of Helsinki and Good Clinical Practice guidelines, and was approved by the institutional ethics review board at each site. The most frequently utilized boards were New England Institutional Review Board, Wellesley, Massachusetts (29 sites in the USA) and IRB Services, Aurora, Ontario (19 sites in Canada). All patients provided written informed consent, which included consent for the extension period.

Patients who completed the first 24-week period were eligible to continue in a long-term, double-blind extension period to 102 weeks. During the extension period, patients remained on their original randomly-assigned (1:1:1:1) blinded treatment (placebo, or dapagliflozin 2.5 mg, 5 mg, or 10 mg) once daily and continued open-label metformin (≥1,500 mg/day). Patients receiving rescue therapy (primarily pioglitazone, or acarbose) during the first 24 weeks continued to receive rescue therapy to 102 weeks. During the 78-week extension period, patients were eligible to receive rescue medication if the HbA1c value was >8.0% during weeks 24 to 50, was >7.5% during weeks 50 to 76, or was >7.0% after week 76. These strict rescue criteria ensured that even with a fully double-blinded long-term design, the participants in all groups were provided with adequate glycemic control. Although the primary endpoint was the change from baseline in HbA1c at 24 weeks [9], the prespecified exploratory objectives in this study included assessment of the change from baseline in HbA1c, fasting plasma glucose (FPG), and body weight in the extension period for each treatment group. The exploratory endpoints also included the proportion of patients who achieved a therapeutic glycemic response defined as HbA1c <7.0% at week 102. Safety outcomes included reported non-serious and serious adverse events, discontinuation due to adverse events, events of special interest, laboratory abnormalities, and change in vital signs as described previously [9].

Formal statistical testing was performed for the primary endpoint and used an analysis of covariance (ANCOVA) model at 24 weeks with the last observation carried forward approach for missing week 24 data excluding data after rescue [9]. At week 102, longitudinal repeated measures analyses using observed data without any data imputation were used to determine the change in HbA1c, FPG, and total body weight from baseline over time; the model included the categorical fixed effects of treatment, week, and treatment-by-week interaction as well as the continuous fixed covariates of baseline measurement and baseline measurement-by-week interaction. Rescue was added as an additional categorical fixed effect in this mixed model when the analysis was performed on data regardless of rescue.

## Results

**Figure 1:**
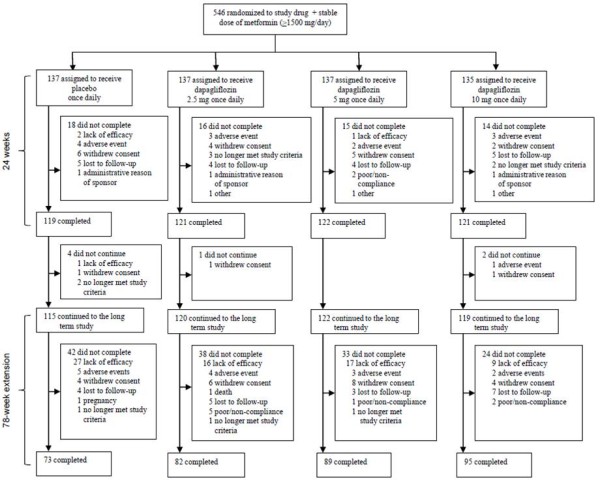
**Trial profile over 102 weeks**.

From the 546 originally randomized patients, 476 (87.2%) continued into the 78-week extension period. Of these, 339 (71.2%) completed the 102-week study (Figure 1). During the 78-week extension, the completion rate was lower for the placebo group (63.5%) than for the dapagliflozin groups (68.3 to 79.8%). This was largely due to more patients in the placebo group (23.5%) withdrawing during the extension period for lack of efficacy, whereas withdrawals were 13.3, 13.9, and 7.6% with dapagliflozin 2.5 mg, 5 mg, and 10 mg, respectively. Demographics and baseline characteristics were balanced between treatment groups as reported previously [9].

**Figure 2:**
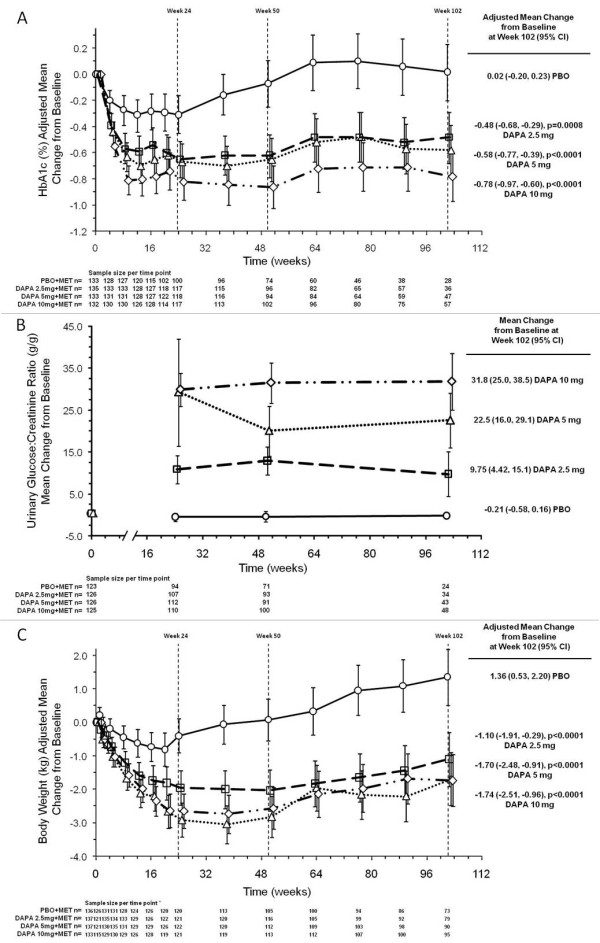
**Change from baseline in (A) glycated hemoglobin (HbA1c), (B) urinary glucose:creatinine ratio, and (C) body weight for placebo (circles), dapagliflozin 2.5 mg (squares), dapagliflozin 5 mg (triangles), and dapagliflozin 10 mg (diamonds) groups all plus metformin up to 102 weeks**. Data are means (95% CI) obtained from longitudinal repeated measures analyses. Data for HbA1c exclude patients who received rescue therapy to achieve the strict HbA1c requirements to remain in the trial. The urinary glucose:creatinine ratios also exclude patients who received rescue therapy. Data for weight, however, include patients who received rescue therapy (pioglitazone), demonstrating the ability of dapagliflozin to attenuate weight gain by this antidiabetes medication. Mean change from baseline in HbA1c after adjustment for baseline value (A), mean change from baseline in urinary glucose:creatinine ratio (g/g) after adjustment for baseline value (B) and mean change from baseline in body weight after adjustment for baseline value (C) are shown.

The mean HbA1c at baseline of all 546 randomized patients was 8.06%. At week 102, the adjusted mean change from baseline in HbA1c was 0.02% (95% CI: -0.20 to 0.23) for placebo and -0.48% (95% CI: -0.68 to -0.29),-0.58% (95% CI: -0.77 to -0.39), and-0.78% (95% CI: -0.97 to -0.60) for dapagliflozin 2.5 mg, 5 mg, and 10 mg, respectively (Table 1). The reductions in HbA1c with dapagliflozin were dose dependent and statistically significant compared to placebo at week 102 (Figure 2A). When excluding data after rescue, 20% (28/137) of patients in the placebo group had observed data for HbA1c at week 102, compared to 26% (36/137), 34% (47/137), and 42% (57/135) of patients receiving dapagliflozin 2.5 mg, 5 mg, and 10 mg, respectively. At week 102, the proportion of patients achieving HbA1c <7% was 20.7 (95% CI: 14.0 to 27.3), 26.4, (95% CI: 19.4 to 33.4), and 31.5% (95% CI: 23.7 to 39.3) for dapagliflozin 2.5 mg, 5 mg, and 10 mg, respectively, vs 15.4% (95% CI: 9.5 to 21.3) for placebo (Table 1); the adjusted proportion of patients achieving HbA1c <7% was statistically significant for dapagliflozin at 5 mg (*P *= 0.0202) and 10 mg (*P *= 0.0014) compared to placebo. Adjusted mean change from baseline in FPG was -0.58 mmol/l (95% CI: -0.97 to -0.19) for placebo and -1.07 mmol/l (95% CI: -1.42 to -0.72), -1.47 mmol/l (95% CI: -1.78 to -1.16), and -1.36 mmol/l (95% CI: -1.65 to -1.07) for the dapagliflozin 2.5 mg, 5 mg, and 10 mg, respectively, at week 102 (Table 1). Adjusted mean change from baseline in FPG was statistically significant for dapagliflozin 5 mg (*P *= 0.0003) and 10 mg (*P *= 0.0012) compared to placebo. The proportions of patients rescued or discontinued for failing to achieve glycemic targets were 60.6% (83/137) for placebo and 51.8% (71/137), 46.0% (63/137), and 42.2% (57/135) for dapagliflozin 2.5 mg, 5 mg, and 10 mg, respectively, at week 102.

**Table 1:** Change from baseline in efficacy parameters

	**Placebo + metformin (n = 137)**	**Dapagliflozin 2.5 mg + metformin (n = 137)**	**Dapagliflozin 5 mg + metformin (n = 137)**	**Dapagliflozin 10 mg + metformin (n = 135)**
**HbA1c (%):^a^**				
Baseline (SD)	8.12 (0.96)	7.99 (0.90)	8.17 (0.96)	7.92 (0.82)
Change at week 24 (95% CI)	-0.31 (-0.45 to -0.16)	-0.65 (-0.79 to -0.51)	-0.67 (-0.81 to -0.53)	-0.82 (-0.96 to -0.68)
Difference vs PBO (95% CI)		-0.35 (-0.55 to -0.14)	-0.36 (-0.56, -0.16)	-0.51 (-0.71, -0.31)
Change at week 102 (95% CI)	0.02 (-0.20 to 0.23)	-0.48 (-0.68 to -0.29)	-0.58 (-0.77 to -0.39)	-0.78 (-0.97 to -0.60)
Difference vs PBO (95% CI)		-0.50 (-079 to -0.21)	-0.60 (-0.89 to -0.31)	-0.80 (-1.08 to -0.52)
*P *vs PBO + MET		0.0008	<0.0001	<0.0001
**FPG (mmol/l):^a^**				
Baseline (SD)	9.19 (2.58)	8.96 (2.39)	9.39 (2.72)	8.66 (2.15)
Change at week 24 (95% CI)	-0.29 (-0.59 to 0.01)	-0.95 (-1.23 to -0.67)	-1.15 (-1.44 to -0.88)	-1.23 (-1.51 to -0.94)
Difference vs PBO (95% CI)		-0.66 (-1.07 to -0.25)	-0.87 (-1.28 to -0.46)	-0.94 (-1.35 to -0.53)
Change at week 102 (95% CI)	-0.58 (-0.97 to -0.19)	-1.07 (-1.42 to -0.72)	-1.47 (-1.78 to -1.16)	-1.36 (-1.65, -1.07)
Difference vs PBO (95% CI)		-0.49 (-0.99 to 0.01)	-0.89 (-1.37 to -0.41)	-0.78 (-1.25 to -0.31)
*P *vs PBO + MET		0.0518	0.0003	0.0012
**Weight (kg):^a^**				
Baseline (SD)	87.74 (19.24)	84.90 (17.77)	84.73 (16.26)	86.28 (17.53)
Change at week 24 (95% CI)	-0.40 (-0.91 to 0.11)	-1.96 (-2.47 to -1.44)	-2.92 (-3.43 to -2.41)	-2.65 (-3.16 to -2.13)
Difference vs PBO (95% CI)		-1.55 (-2.27 to -0.84)	-2.52 (-3.23 to -1.80)	-2.24 (-2.96 to -1.53)
Change at week 102 (95% CI)	1.36 (0.53 to 2.20)	-1.10 (-1.91 to -0.29)	-1.70 (-2.48 to -0.91)	-1.74 (-2.51 to -0.96)
Difference vs PBO (95% CI)		-2.46 (-3.63 to -1.30)	-3.06 (-4.21 to -1.92)	-3.10 (-4.24 to -1.96)
*P *vs PBO + MET		<0.0001	<0.0001	<0.0001
**Percentage of patients with HbA1c <7.0% (95% CI):^b^**				
At week 24	24.2 (17.4 to 30.9)	30.9 (23.4 to 38.4)	35.9 (28.4 to 43.5)	38.2 (30.3 to 46.1)
Difference vs PBO (95% CI)		6.7 (-3.2 to 16.6)	11.8 (1.9 to 21.6)	14.0 (3.9 to 24.1)
At week 102	15.4 (9.5 to 21.3)	20.7 (14.0 to 27.3)	26.4 (19.4 to 33.4)	31.5 (23.7 to 39.3)
Difference vs PBO (95% CI)		5.3 (-3.5 to 14.1)	11.0 (1.9 to 20.0)	16.1 (6.4 to 25.7)
*P *vs PBO + MET		0.2380	0.0176	0.0011

n indicates the number of randomized patients who took at least one dose of double-blind study medication. Data are means (SD) or means (95% CI). ^a^Adjusted mean change from baseline using longitudinal repeated measures, excluding data after rescue, except weight, which includes data after rescue. ^b^Percentage adjusted for baseline glycated hemoglobin (HbA1c). Patients discontinued or rescued prior to week t (t = 24 or 102) or missed measurements at week t are considered not achieving glycemic response. DAPA = dapagliflozin; FPG = fasting plasma glucose; MET = metformin; PBO = placebo.

As shown in Figure 2B, large increases in the urinary glucose:creatinine ratio for the dapagliflozin groups were dose dependent and maintained through to week 102. For total body weight, when excluding data after rescue therapy, the adjusted mean value decreased from baseline with dapagliflozin (-2.16 kg to -3.38 kg) and with placebo (-0.67 kg) through week 102. When including data after rescue, recognizing that pioglitazone was the primary rescue medication, the adjusted mean value still decreased from baseline with dapagliflozin (-1.10 kg to -1.74 kg) but increased with placebo (1.36 kg) through week 102, and differences between the dapagliflozin groups versus placebo were statistically significant at 102 weeks (Table 1, Figure 2C).

The proportions of patients who reported at least one adverse event were similar across dapagliflozin groups and the placebo group when including data after rescue (Table 2). During the 78-week extension, three deaths occurred (Table 2): two in the dapagliflozin 2.5 mg group due to cardiopulmonary arrest and myocardial infarction, and one death in the placebo group due to malignant lung neoplasm. All three deaths were reported to be unlikely related or not related to the study treatment as assessed by the investigator. The proportions of serious adverse events through 102 weeks were similar across dapagliflozin groups and the placebo group (Table 2). Few patients reported adverse events that led to discontinuation, and the proportion was similar in all treatment groups (Table 2). No major episodes of hypoglycemia occurred, and no discontinuations were due to hypoglycemia. The proportion of patients who reported at least one event of hypoglycemia was similar across study groups (Table 2).

**Table 2:** Adverse and special interest events up to week 102

	**Placebo + metformin**	**Dapagliflozin 2.5 mg + metformin**	**Dapagliflozin** **5 mg + metformin**	**Dapagliflozin 10 mg + metformin**
N	137	137	137	135
At least one adverse event	111 (81.0)	111 (81.0)	111 (81.0)	111 (82.2)
At least one related adverse event	28 (20.4)	36 (26.3)	33 (24.1)	45 (33.3)
Adverse event leading to discontinuation	9 (6.6)	7 (5.1)	5 (3.6)	6 (4.4)
At least one serious adverse event	14 (10.2)	15 (10.9)	9 (6.6)	14 (10.4)
Deaths	1 (0.7)	2 (1.5)	0	0
**Most common adverse events (>10% in any treatment group):**				
Back pain	11 (8.0)	11 (8.0)	7 (5.1)	18 (13.3)
Influenza	15 (10.9)	19 (13.9)	20 (14.6)	17 (12.6)
Diarrhea	10 (7.3)	7 (5.1)	9 (6.6)	16 (11.9)
Urinary tract infection	8 (5.8)	6 (4.4)	8 (5.8)	16 (11.9)
Headache	8 (5.8)	10 (7.3)	13 (9.5)	15 (11.1)
Nasopharyngitis	12 (8.8)	16 (11.7)	8 (5.8)	12 (8.9)
Upper respiratory tract infection	14 (10.2)	9 (6.6)	5 (3.7)	5 (3.7)
**Adverse events of special interest:**				
Suggestive of urinary tract infection	11 (8.0)	11 (8.0)	12 (8.8)	18 (13.3)
Experiencing a single event	8/11 (72.7)	8/11 (72.7)	10/12 (83.3)	11/18 (61.1)
Men	3/76 (3.9)	2/70 (2.9)	2/69 (2.9)	4/77 (5.2)
Women	8/61 (13.1)	9/67 (13.4)	10/68 (14.7)	14/58 (24.1)
Suggestive of genital infection	7 (5.1)	16 (11.7)	20 (14.6)	17 (12.6)
Experiencing a single event	7/7 (100)	15/16 (93.8)	16/20 (80.0)	14/17 (82.4)
Men	0/76	4/70 (5.7)	4/69 (5.8)	5/77 (6.5)
Women	7/61 (11.5)	12/67 (17.9)	16/68 (23.5)	12/58 (20.7)
Renal impairment or failure	2 (1.5)	6 (4.4)	4 (2.9)	2 (1.5)
**Hypoglycemia:**				
Total patients with hypoglycemia	8 (5.8)	5 (3.6)	7 (5.1)	7 (5.2)
Major episode of hypoglycemia^a^	0 (0)	0 (0)	0 (0)	0 (0)
Hypotension, dehydration, hypovolemia	2 (1.5)	0 (0)	3 (2.2)	2 (1.5)

Data are n (%) or n out of N (%); includes data after rescue. ^a^Major episode defined as a symptomatic episode requiring external assistance due to severe impairment in consciousness or behavior with a capillary or plasma glucose value <3 mmol/l and prompt recovery after glucose or glucagon administration.

As shown in Table 2, signs, symptoms, and other evidence suggestive of urinary tract infection (UTI) occurred at a higher rate in the dapagliflozin 10 mg group (13.3%) compared to placebo (8.0%), dapagliflozin 2.5 mg group (8.0%), and dapagliflozin 5 mg group (8.8%), with one discontinuation (dapagliflozin 2.5 mg). Signs, symptoms and other evidence suggestive of vulvovaginitis, balanitis, and related genital infection (not sexually transmitted) were more common in the dapagliflozin groups (11.7% to 14.6%) than placebo (5.1%) (Table 2) with one discontinuation (dapagliflozin 5 mg). Evidence suggestive of UTI or genital infection occurred more frequently in women than in men. These events were mild or moderate in intensity, with >65% occurring in the first 24 weeks. Signs or symptoms suggestive of UTI or genital infection responded to standard treatment typically without interruption of dapagliflozin therapy and rarely led to recurrence. No events of pyelonephritis were reported.

The proportions of renal impairment or failure events (defined by a prespecified list) were higher with dapagliflozin in total than with placebo, but similar proportions between dapagliflozin 10 mg and placebo were reported (Table 2). Of the 14 events (in all groups) of renal impairment or failure, 8 consisted of increases in serum creatinine ≥1.5 times the baseline value, or attaining an absolute value of 221 μmol/l. One patient in the dapagliflozin 5 mg group had a serious adverse event of acute renal failure at day 624 due to urinary obstruction that resulted in discontinuation and resolved following prostatectomy. Adverse events of hypotension, or suggestive of dehydration and hypovolemia were infrequent, non-severe, and similar between the placebo and dapagliflozin groups (Table 2). Fractures were reported by two patients (1.5%) each in the placebo, dapagliflozin 2.5 mg, and dapagliflozin 5 mg groups, and by three patients (2.2%) in the dapagliflozin 10 mg group during the 102-week study. One patient on dapagliflozin 5 mg with a history of hematuria that predated randomization experienced a bladder transitional cell cancer. One patient on dapagliflozin 10 mg was diagnosed with breast cancer within the first year of enrollment.

As shown in Table 3, no clinically relevant mean changes from baseline in sodium, potassium, creatinine or blood urea nitrogen were apparent in any treatment group through week 102. Mean decreases from baseline in uric acid (46 to 56 μmol/l) were noted in all dapagliflozin groups and were significantly greater versus placebo. Small mean increases in hemoglobin and hematocrit, which were seen by about week 12 with dapagliflozin, remained stable thereafter. Although mean systolic and diastolic blood pressure decreased by 2.1 to 5.1 mmHg and 1.8 to 2.5 mmHg, respectively, at 24 weeks in patients receiving dapagliflozin (2.5 mg, 5 mg, and 10 mg) versus 0.2 and 0.1, respectively, for placebo [9], smaller decreases from baseline were observed at 102 weeks in mean systolic and diastolic blood pressure with dapagliflozin therapy.

**Table 3:** Summary of laboratory parameters

	**Placebo + metformin (n = 137)**	**Dapagliflozin 2.5 mg + metformin (n = 137)**	**Dapagliflozin 5 mg + metformin (n = 137)**	**Dapagliflozin 10 mg + metformin(n = 135)**
**Sodium (mmol/l)**				
N	72	77	87	93
Baseline	138.9 (2.3)	138.5 (2.9)	138.6 (2.3)	139.1 (2.2)
Change at week 102	-0.04 [2.7]	0.2 [2.8]	0.1 [2.6]	0.1 [2.6]
*P *vs PBO + MET		0.1434	0.1905	0.3978
**Potassium (mmol/l)**				
n	72	77	87	93
Baseline	4.35 (0.47)	4.34 (0.46)	4.37 (0.39)	4.37 (0.43)
Change at week 102	-0.08 [0.55]	-0.01 [0.45]	-0.05 [0.35]	-0.07 [0.47]
*P *vs PBO + MET		0.3366	0.4563	0.9918
**Serum creatinine (μmol/l)**				
n	72	77	88	93
Baseline	76.9 (17.9)	78.7 (18.1)	79.6 (19.3)	77.8 (17.8)
Change at week 102	-0.9 [10.3]	-1.8 [9.7]	-3.5 [12.7]	-2.7 [10.6]
*P *vs PBO + MET		0.9954	0.4720	0.9032
**Serum uric acid (μmol/l)^a^**				
n	28	35	47	57
Baseline	314 (79)	322 (80)	323 (88)	323 (80)
Change at week 102	-1.78 [54.13]	-55.9 [50.32]	-46.4 [64.36]	-52.9 [64.36]
*P *vs PBO + MET		0.0054	0.0013	0.0002
**Blood urea nitrogen (mmol/l)**				
n	72	77	88	93
Baseline	5.33 (1.55)	5.51 (1.41)	5.65 (1.81)	5.34 (1.41)
Change at week 102	0.56 [1.24]	0.70 [1.54]	0.42 [1.87]	0.73 [1.44]
*P *vs PBO + MET		0.2299	0.4169	0.0845
**Hematocrit (%)**				
n	71	78	87	93
Baseline	42.61 (3.90)	42.38 (3.99)	42.15 (3.59)	42.88 (3.95)
Change at week 102	-1.43 [3.29]	0.84 [2.53]	1.35 [2.48]	1.54 [2.78]
*P *vs PBO + MET		<0.0001	<0.0001	<0.0001
**Hemoglobin (g/l)**				
n	71	78	87	94
Baseline	142.8 (13.45)	142.5 (14.25)	141.6 (12.19)	143.6 (13.10)
Change at week 102	-4.9 [10.14]	1.5 [7.73]	3.1 [8.40]	4.1 [8.84]
*P *vs PBO + MET		<0.0001	<0.0001	<0.0001
**Systolic blood pressure (mmHg)**				
n	72	78	88	94
Baseline	128 (15)	127 (14)	127 (14)	126 (16)
Change at week 102	1.5 [13.7]	0.7 [16.1]	-1.1 [13.2]	-0.3 [15.0]
*P *vs PBO + MET		0.1111	0.0136	0.0067
**Diastolic blood pressure (mmHg)**				
n	72	78	88	94
Baseline	81 (9)	80 (9)	81 (9)	79 (10)
Change at week 102	-1.0 [7.9]	-0.1 [8.1]	-1.5 [8.1]	-1.2 [10.1]
*P *vs PBO + MET		0.6605	0.2140	0.1075

Data are means (SD) at baseline or mean changes [SE] from baseline at week 102 including data after rescue unless noted. n is the number of treated patients with non-missing baseline and week 102 values for the specific test. ^a^Excluding data after rescue. DAPA = dapagliflozin; MET = metformin; PBO = placebo.

## Discussion

In the present long-term study, dapagliflozin demonstrated durability of glycemic and weight-loss benefits for 102 weeks when added to metformin in patients with type 2 diabetes who were inadequately controlled on metformin alone, thus indicating that dapagliflozin may be used for long-term management of excess HbA1c and body weight. This treatment acts independently of β-cell function or insulin sensitivity, thereby suggesting an alternative or complementary approach to currently available treatments and offering an additional therapeutic option throughout the natural history of type 2 diabetes. Recent approval by the European Medicines Agency makes dapagliflozin the first agent in this new class of SGLT2 inhibitors.

Strengths of this study include its design as a large trial that remained double-blinded throughout the long-term extension, unlike those conducted as open-label extensions. A greater proportion of dapagliflozin-treated patients remained in the trial throughout the full 2 years, substantiating the durability of the glucose-lowering effect. There were no apparent baseline differences across study groups to explain the variation in glycemic outcomes; however, treatment-related weight reduction is likely to be a factor in enhancement of glucose reduction. While efficacy analyses generally excluded rescue, safety analyses included all data regardless of rescue in order to get a comparison to placebo that was as unbiased as possible. A potential limitation of this study is the number of patients needing rescue medication in the placebo group. Although this might limit the statistical interpretation of durability of the glucose-lowering effect of dapagliflozin, it also emphasizes the clinical utility of dapagliflozin. The present study design, which included a placebo control group for 102 weeks, employed strict glycemic control criteria to ensure that all patients received a quality of care consistent with current guidelines for the treatment of type 2 diabetes. Thus, patients whose HbA1c exceeded 7.5% at 50 weeks or 7.0% at 76 weeks received rescue therapy and were not included in the final efficacy analysis. Failure to maintain a consistent decrease in blood pressure during long-term dapagliflozin therapy may reflect the extension trial design in which investigators could adjust antihypertensive therapy according to clinical need, which may have overridden any potential blood pressure lowering effect from dapagliflozin. In addition, the mean baseline blood pressure was already near or at goal for patients with diabetes.

To date, the published clinical trial data on dapagliflozin are from short-term (12 to 24 weeks) [6,8-10] or 1-year trials [11,13,14], all of which have demonstrated efficacy of this agent in management of hyperglycemia. The data from this 102-week trial demonstrate sustained glycemic and weight-loss benefits. Because SGLT2 inhibition is an investigative and relatively new concept within the type 2 diabetes arena, no direct comparative data with other SGLT2 agents are available. Dapagliflozin has demonstrated greater or augmented efficacy compared with commonly prescribed antidiabetic medications [6,8-11,13,14], suggesting this novel mechanism of action may be considered as an addition to treatment options for patients with type 2 diabetes for its advantage of having a sustained effect in HbA1c reduction and weight loss.

The durability of type 2 diabetes therapy is typically limited by the natural history of the disease such that the progressive decline in β-cell function superimposed upon insulin resistance restricts the continuing efficacy of interventions that are dependent on insulin production or insulin action [1]. The present study demonstrates the increased glycemic and weight-loss benefits of the non-insulin-dependent SGLT2 inhibition mechanism. The low rate of discontinuations due to adverse events indicates a favorable tolerability profile that makes this agent an important addition to the treatment armamentarium. As this is the first entry in a new class of antidiabetic agents, dapagliflozin provides an important additional option for clinicians managing patients with poorly controlled type 2 diabetes.

A better understanding of the relationship between glucosuria and genitourinary infections should be provided with ongoing safety analyses of the dapagliflozin trial program. Further study of the effects of dapagliflozin on metabolic surrogate markers will assist in discrimination of non-glycemic benefits. As an example, increased serum uric acid is frequently associated with raised BMI, blood pressure, FPG, and triglycerides [15,16] and has been implicated as part of the 'metabolic syndrome' cluster of risk factors for diabetes and cardiovascular complications [17,18]. The mean decreases in serum uric acid observed with dapagliflozin here and in other trials [5,6], which are consistent with the uricosuric action of this agent, might therefore indicate potential added benefits, but this requires further investigation.

## Conclusions

Dapagliflozin added to metformin over 102 weeks showed sustained improved glycemic control, modest weight reduction, and no increased risk of hypoglycemia in type 2 diabetes inadequately controlled with metformin alone. These effects suggest a complementary use of the insulin independent effects of dapagliflozin in combination with metformin.

## Abbreviations

FPG: fasting plasma glucose; HbA1c: glycated hemoglobin; SGLT2: sodium-glucose cotransporter 2; UTI: urinary tract infection.

## Competing interests

All authors have completed the Unified Competing Interest form at http://www.icmje.org/coi_disclosure.pdf (available upon request from the corresponding author). CJB has attended advisory board meetings of Bristol-Myers Squibb and AstraZeneca; undertaken ad-hoc consultancy for Bristol-Myers Squibb, AstraZeneca, Merck Sharp & Dohme, Novo Nordisk, GlaxoSmithKline, Eli Lilly, and Takeda; received research grants from AstraZeneca and Sanofi-Aventis; delivered continuing medical education programs sponsored by Bristol-Myers Squibb, AstraZeneca, GlaxoSmithKline, Merck Serono, and Merck Sharp & Dohme; and received travel or accommodations reimbursement from GlaxoSmithKline and Bristol-Myers Squibb. JLG, a trialist for this study, has attended advisory board meetings, received research support, and undertaken clinical trials sponsored by Bristol-Myers Squibb. DH is an employee of Bristol-Myers Squibb. NI, TAM, and JFL are employees and shareholders of Bristol-Myers Squibb.

## Authors' contributions

CJB and JLG oversaw development of the manuscript. All authors made substantial contributions to conception and design, acquisition of data or analysis and interpretation of data, and revising the article for important content. All authors approved the final version of the manuscript.

## Pre-publication history

The pre-publication history for this paper can be accessed here:

http://www.biomedcentral.com/1741-7015/11/43/prepub
