# Correction: Dapagliflozin add-on to metformin in type 2 diabetes inadequately controlled with metformin: a randomized, double-blind, placebo-controlled 102-week trial

**DOI:** 10.1186/1741-7015-11-193

**Published:** 2013-09-02

**Authors:** Clifford J Bailey, Jorge L Gross, Delphine Hennicken, Nayyar Iqbal, Traci A Mansfield, James F List

**Affiliations:** 1 Life and Health Sciences, Aston University, Birmingham B4 7ET, UK; 2 Endocrine Division, Universidade Federal do Rio Grande do Sul, Avenida Paulo Gama 110-Reitoria, Porto Alegre 90040-060, Brazil; 3 Global Biometric Services, Bristol-Myers Squibb, Parc de l’Alliance, Avenue de Finlande 4, Braine I’Alleud B-1420, Belgium; 4 Global Clinical Research, Bristol-Myers Squibb, Route 206 & Province Line Road, Princeton, NJ 08543, USA

## Correction

The authors [1] note that the standard errors in Table 3 (Table 1 here) are actually standard deviations. The reference to standard errors in the footnote has now been edited accordingly.

This is a Correction article on http://www.biomedcentral.com/1741-7015/11/43.

**Table 1:** Summary of laboratory parameters

	**PBO + MET (n = 137)**	**DAPA 2.5 mg + MET (n = 137)**	**DAPA 5 mg + MET (n = 137)**	**DAPA 10 mg + MET (n = 135)**
**Sodium (mmol/L)**				
n	72	77	87	93
Baseline	138.9 (2.3)	138.5 (2.9)	138.6 (2.3)	139.1 (2.2)
Change at week 102	-0.04 (2.7)	0.2 (2.8)	0.1 (2.6)	0.1 (2.6)
*P* vs PBO + MET		0.1434	0.1905	0.3978
**Potassium (mmol/L)**				
n	72	77	87	93
Baseline	4.35 (0.47)	4.34 (0.46)	4.37 (0.39)	4.37 (0.43)
Change at week 102	-0.08 (0.55)	-0.01 (0.45)	-0.05 (0.35)	-0.07 (0.47)
*P* vs PBO + MET		0.3366	0.4563	0.9918
**Serum creatinine (μmol/L)**				
n	72	77	88	93
Baseline	76.9 (17.9)	78.7 (18.1)	79.6 (19.3)	77.8 (17.8)
Change at week 102	-0.9 (10.3)	-1.8 (9.7)	-3.5 (12.7)	-2.7 (10.6)
*P* vs PBO + MET		0.9954	0.4720	0.9032
**Serum uric acid (μmol/L)**^**a**^				
n	28	35	47	57
Baseline	314 (79)	322 (80)	323 (88)	323 (80)
Change at week 102	-1.78 (54.13)	-55.9 (50.32)	-46.4 (64.36)	-52.9 (64.36)
*P* vs PBO + MET		0.0054	0.0013	0.0002
**Blood urea nitrogen (mmol/L)**				
n	72	77	88	93
Baseline	5.33 (1.55)	5.51 (1.41)	5.65 (1.81)	5.34 (1.41)
Change at week 102	0.56 (1.24)	0.70 (1.54)	0.42 (1.87)	0.73 (1.44)
*P* vs PBO + MET		0.2299	0.4169	0.0845
**Haematocrit (%)**				
n	71	78	87	93
Baseline	42.61 (3.90)	42.38 (3.99)	42.15 (3.59)	42.88 (3.95)
Change at week 102	-1.43 (3.29)	0.84 (2.53)	1.35 (2.48)	1.54 (2.78)
*P* vs PBO + MET		<0.0001	<0.0001	<0.0001
**Haemoglobin (g/L)**				
n	71	78	87	94
Baseline	142.8 (13.45)	142.5 (14.25)	141.6 (12.19)	143.6 (13.10)
Change at week 102	-4.9 (10.14)	1.5 (7.73)	3.1 (8.40)	4.1 (8.84)
*P* vs PBO + MET		<0.0001	<0.0001	<0.0001
**Systolic blood pressure (mm Hg)**				
n	72	78	88	94
Baseline	128 (15)	127 (14)	127 (14)	126 (16)
Change at week 102	1.5 (13.7)	0.7 (16.1)	-1.1 (13.2)	-0.3 (15.0)
*P* vs PBO + MET		0.1111	0.0136	0.0067
**Diastolic blood pressure (mm Hg)**				
n	72	78	88	94
Baseline	81 (9)	80 (9)	81 (9)	79 (10)
Change at Week 102	-1.0 (7.9)	-0.1 (8.1)	-1.5 (8.1)	-1.2 (10.1)
*P* vs PBO + MET		0.6605	0.2140	0.1075

Data are means (SD) at baseline or mean changes (SD) from baseline at week 102 including data after rescue unless noted. n is the number of treated patients with non-missing baseline and Week 102 values for the specific test. ^**a**^Excluding data after rescue. Abbreviations: *SD* standard deviation, *SE* standard error, *PBO* placebo, *MET* metformin, *DAPA* dapagliflozin.

## Pre-publication history

The pre-publication history for this paper can be accessed here:

http://www.biomedcentral.com/1741-7015/11/193/prepub
